# Impact of hyperfractionated re-irradiation on quality of life in patients with recurrent or second primary head and neck cancer, a prospective single institutional study

**DOI:** 10.1016/j.ctro.2023.100654

**Published:** 2023-06-22

**Authors:** Cecilie Delphin Amdal, Jon Magne Moan, Einar Dale, Ragnhild Sørum Falk, Safora Johansen, Kristin Bjordal

**Affiliations:** a Department of Oncology, Oslo University Hospital, Oslo, Norway; b Research Support Services, Oslo University Hospital, Oslo, Norway; c Oslo Metropolitan University, Faculty of Health Sciences, Oslo, Norway; d University of Oslo, Faculty of Medicine, Oslo, Norway

**Keywords:** Head and neck cancer, Recurrent disease, Radiotherapy, Health-related quality of life, Patient-reported outcome measure

## Abstract

**Purpose:**

Describe the clinical outcome of hyperfractionated re-irradiation (HFRT) in patients with recurrent or second primary (SP) head and neck cancer (HNC).

**Methods:**

This prospective observational study included HNC patients eligible for HFRT. Inclusion criteria: age ≥18 years, recurrent or SP HNC, planned re-irradiation and ability to respond to questionnaires. Patients received 1.5 Gy twice daily, five days a week for three (palliative) or four (curative/local control) weeks, total dose 45/60 Gy. Toxicity was scored with CTCAE v3 at baseline, end of treatment, at three, six, 12 and 36 months follow-up. Health-related quality of life (HRQoL) was measured with EORTC QLQ-C30 and EORTC QLQ-H&N35, pre-treatment and eight times until 36 months. In the main outcome (Global quality of life and H&N Pain), a change score of ≥10 was considered clinically significant, and p-values < 0.05 (two-sided) statistically significant. The Kaplan-Meier method was used for survival analyses.

**Results:**

Over four years from 2015, 58 patients were enrolled (37 recurrent and 21 SP). All, but two patients completed treatment as planned. Toxicity (≥grade 3) increased from pre-treatment to end of treatment with improvement in the follow-up period. The mean Global quality of life (QoL) and H&N Pain scores were stable from pre-treatment to three months. Maintained/ improved Global QoL was reported by 60% of patients at three months and 56% of patients at 12 months. For patients with curative, local control and palliative intent, the median survival (range) was 23 (2–53), 10 (1–66) and 14 (3–41) months respectively. Of those alive, the proportion of disease-free patients at 12 and 36 months, were 58% and 48%, respectively.

**Conclusion:**

Most HNC patients reported maintained HRQoL at three and 12 months after HFRT despite serious toxicity observed in many patients. Long-term survival can be achieved in a limited proportion of the patients.

## Highlights

• QoL maintained after re-irradiation in patients with relapsed head and neck cancer.
• Hyperfractionated re-irradiation was feasible for head and neck cancer patients.
• High prevalence of toxicity at end of treatment.
• The majority of patients alive at one and three years had received ≥60 Gy.

## 1 Introduction

Around 30–50% of patients with locally advanced head and neck cancer (HNC) experience recurrence of disease within the first five years [1]. The life-long risk of developing second primary (SP) HNC is around 20% [2]. A reduced life expectancy is observed for both groups [3]. Fewer than 20% of patients with recurrent HNC live five years or more [4]. They often experience multiple symptoms, functional problems and reduced health-related quality of life (HRQoL) due to their disease, previous and current treatment. Systematic information on patient-reported outcomes (PRO) before, during and after re-irradiation is limited [5], [6], [7].

Patients with recurrent or SP HNC that have inoperable disease or with non-radical resections are candidates for re-irradiation [8], [9]. The scientific community has discussed potential benefits of re-irradiation for many years [8], [10], [11], without reaching a consensus on the optimal treatment regimen. Usually, 60 Gy is needed to achieve local control [9] and typical regiments used are; conventional fractionated regimens (2 Gy per fraction, 5 fractions a week, total dose ≥50 Gy) with or without concomitant chemotherapy [12] or hyperfractionated radiotherapy (HFRT) regimens (1.2 to 1.5 Gy twice daily, 10 fractions a week, total dose 60 Gy) with or without concomitant chemotherapy [13]. The HFRT has been applied in order to increase the therapeutic ratio [10].

HFRT is demanding when applied to a small group of vulnerable patients with poor prognosis. Evaluation of the benefit and burden of such treatment is essential. The lack of systematic information about these patients’ symptoms, side effects and general well-being before, during and after treatment prompted this study. We aimed to assess HRQoL, tolerability (feasibility and toxicity), and survival outcome of HFRT in patients with recurrent or SP HNC.

## 2 Materials and methods

### 2.1 Design and patients

This sub-study is part of a larger prospective, longitudinal observational cohort study titled “Re-Irradiation or Other Treatment of patients with Head and Neck cancer unsuited for radical radiotherapy, evaluation with patient reported outcomes (RIOT H&N)” conducted at Oslo University Hospital (OUH). The aim of this study was to gain more knowledge about clinical outcomes (survival and HRQoL) of treatment in this patient group. We aimed to include 200 patients within a maximum period of four years. Patients with primary HNC unfit for surgery or radical RT, or with metastatic disease, second primary or recurrent disease, were eligible for inclusion. Inclusion criteria were verified invasive carcinoma in the head and neck region, age ≥18 years, with ability to respond to questionnaires. Exclusion criteria were planned standard curative treatment (radical RT +/-concomitant chemotherapy or postoperative RT), or previous re-irradiation. In the multi-disciplinary team (MDT) meeting, all patients were discussed and the treatment decision (HFRT, hypo fractionated RT, chemotherapy, other) was made. Re-irradiation was not recommended within six months after primary RT (but was not a study exclusion criteria) and for patients with RT-related severe trismus, ulcerations or osteoradionecrosis. Necessary approvals were obtained and participants provided written informed consent. In this sub-study, all patients planned for re-irradiation were included. The other patient groups will be analysed in another sub-study. For patients receiving re-irradiation, overlap with the previous planning tumour volume (PTV) was not an exclusion criterion.

### 2.2 Treatment

The patients performed contrast-enhanced planning CT or PET/CT in treatment position with a thermoplastic mask and neck support. Targets and organs at risk (OARs) were delineated on the CT or PET/CT and the treatment planning was performed according to standard procedures [14] using IMRT (exceptionally three dimensional conformal treatment planning) in RayStation® (v4.5/5.0, RaySearch Laboratories, Stockholm; Sweden). In inoperable patients, the clinical target volume (CTV) included the gross tumour volume (GTV) with 5 mm margin. Post-surgery, the CTV covered the original tumour volume with ≥10 mm margin including the surgical bed. PTV was generated with 3 mm margin to the CTV with the target of achieving minimum 95% of the prescribed dose to 98% of the volume (D98_95%).

HFRT was previously applied over a five-week period with one-week intermission after two weeks to increase tolerability [10]. From 2014, the intermission was omitted to increase the therapeutic ratio. The new regimen was 1.5 Gy per fractions twice daily (minimum six hours apart), five days a week for three (palliative) or four (curative, local control) weeks with total dose of 45 or 60 Gy. Some patients participated in a dose painting sub-study with total dose 70–75 Gy to a sub-volume within the GTV [15].

### 2.3 Clinical data collection and HRQoL assessments

The patients received follow-up consultation at the out-patient clinic according to guidelines; at pre-treatment, end of treatment and during follow-up. The follow-up of this patient group is focused on symptom relief and HRQoL avoiding examinations without clinical implication. At pre-treatment, sociodemographic and clinical data, including comorbidity according to Charlson’s co-morbidity index [16] were collected (Table 1). WHO performance status (PS), weight, nutritional support, use of analgesics and observer-rated toxicity according to the Common Terminology Criteria for Adverse Events (CTCAE v3.0) [17] were also collected at end of treatment (EOT) and at three, six, 12 and 36 months. Disease status (tumour free, not tumour free, relapse suspected, and unavailable) was assessed at the follow-up visits based on clinical and/ or radiological evaluation. The time and cause of death was collected from medical records.

**Table 1:** Characteristics of patients with relapse of head and neck cancer or second primary in the head and neck area (n = 58).

Characteristics		mean [median] (range) or n (%)
Age (years)		64.5 [66.5] (22–89)
Gender	Male	42 (72)
	Female	16 (28)
Disease status	Relapse/progression	37 (64)
	Second primary (SP)	21 (36)
	TNM stage* I-III	8 (14)
	TNM stage* IV	13 (22)
Tumour location	Oral cavity	25 (43)
	Oropharynx	15 (26)
	Hypopharynx	7 (12)
	Nasopharynx	1 (2)
	Larynx	6 (10)
	Other (Sinonasal, salivary gland, unknown primary)	4 (7)
Histology	Squamous cell carcinoma	52 (90)
	Adenocarcinoma	2 (3)
	Other	4 (7)
HPV status	Positive	8 (14)
	Negative	6 (10)
	Unknown	1 (2)
	Not applicable**	43 (74)
WHO performance status	0	17 (29)
	1	29 (50)
	2	12 (21)
	3–4	0 (0)
Charlson’s co-morbidity index	0	32 (55)
	1	12 (21)
	≥ 2	14 (24)
Weight loss last six months	≤ 5 %	36 (62)
	> 5 %	21 (36)
	Unknown	1 (2)
Smoking history	Never	15 (26)
	Stopped	25 (43)
	Ongoing	18 (31)
	≤ 20 pack years	19/43
	> 20 pack years	18/43
	Pack years missing	6/43
Alcohol history	Never or occasionally	24 (41)
	Weekly	22 (38)
	Daily	10 (17)
	Unknown	2 (3)
Enteral nutritional support	No	48 (83)
	Nasogastric tube	2 (3)
	Percutaneous endoscopic gastrostomy	8 (14)
Opioid (regular or on demand)	No	30 (52)
	Yes	28 (48)

* Stage TNM 8th edition.

* HPV status not applicable for other than oropharyngeal cancer.

The patients completed validated Norwegian questionnaires at pre-treatment, EOT, three, six and every six months until 36-month follow-up at the hospital or at home. The European Organisation for Research and Treatment of Cancer (EORTC) quality of life core questionnaire (QLQ-C30) and HNC specific module, the EORTC QLQ-H&N35 [18], contains function and symptom scales and single items. Responses were given on a four-point Likert scales ranging from 1 (not at all) to 4 (very much) or a modified visual analogue scale ranging from 1 (very poor) to 7 (excellent). All answers were converted to 0–100 scales where high score represents high level of functioning or high level of symptoms.

### 2.5 Statistical methods

Descriptive analyses are presented as frequencies and proportions for categorical data, and means with standard deviations (SD), and medians and range for continuous data. The main endpoints were change in QLQ-C30 global health status / QoL scale (Global QoL) and H&N specific Pain (H&N Pain) from pre-treatment to three and 12 months follow-up. Change was presented as mean change with 95% confidence interval (CI) and as the proportion of patients with maintained (change < ±10) or improved (change ≥ 10) HRQoL. For the HRQoL variables, a change of ≥ 10 was regarded as clinically significant [19]. Paired *t*-test was used to compare the mean HRQoL scores within patients over time. P-values < 0.05 (two-sided) were regarded as statistically significant. Missing items were handled as recommended [20] without imputation. The median overall and one and three-year survival rates were assessed. The patients were followed from the time of diagnosis of the recurrence or SP to time of death of any cause or censored if alive at the end of study (December 15th 2021). No patients were lost to follow-up. We used the Kaplan-Meier method to calculate survival. Statistical analyses were performed in SPSS® v26 and Stata® v16.

## 3 Results

### 3.1 Patients and treatment

**Fig. 1:**
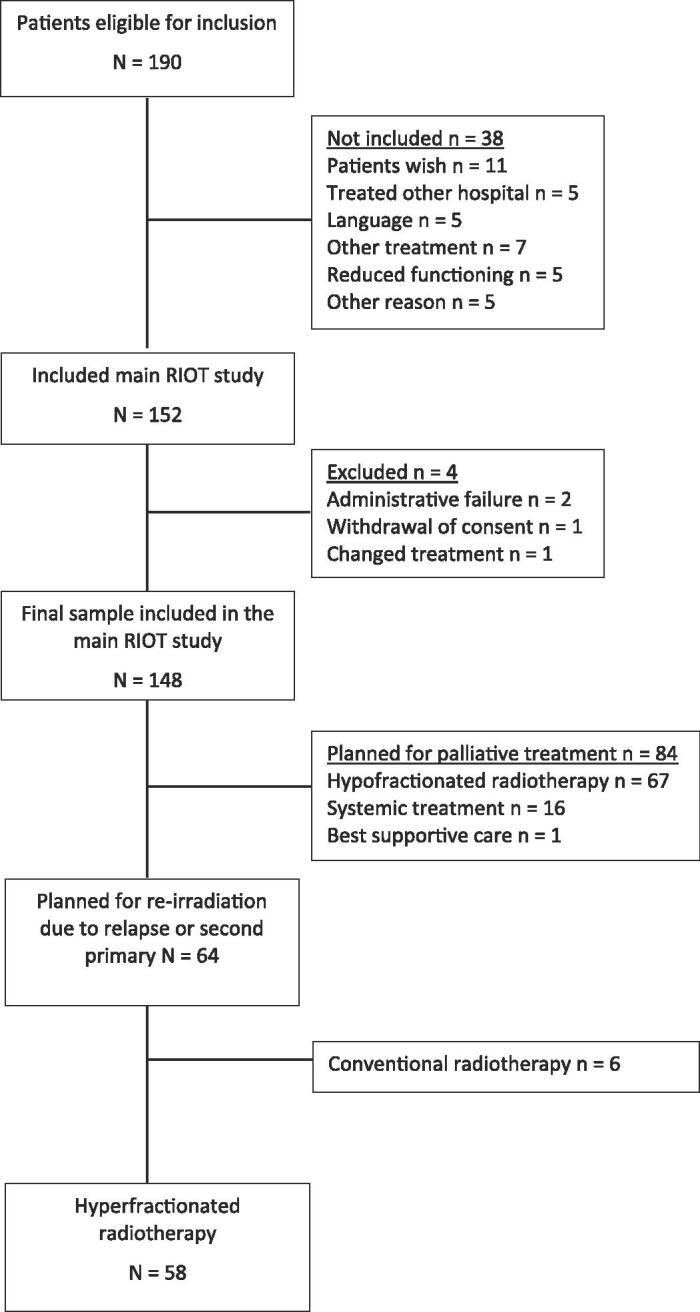
Flow chart, inclusion in RIOT and the sub-study of patients eligible for hyperfractionated re-irradiation.

Of 190 eligible patients, 152 were included in RIOT H&N within the four-year period from June 2015, 58 of these patients were candidates for re-irradiation and they were included in this sub-study (Fig. 1). All these 58 patients were treated with HFRT; 37 (64%) of them had relapse of disease and 21 (36%) had SP HNC (Table 1). Most patients had squamous cell carcinoma of the oral cavity (43%), good PS (79%), and no or low burden of comorbidity (76%). For SPs, 10/21 tumours were located in the same anatomical area as the previous primary tumour. The mean time since primary RT was six years, with a large variation (five months to 27 years) (Table 2). The two patients who received re-irradiation within six months had relapse of disease just outside the margin of the previous RT field with complete response within the margin and had no serious RT related toxicity (grade ≤ 2).

**Table 2:** Treatment characteristics of patients with relapse of head and neck cancer or second primary in the head and neck area (n = 58).

**Characteristics**		**mean [median] (range) or n (%)**
Time since previous radiotherapy (years)		6 [3] (0–27)
Time since previous radiotherapy	< 6 months	2 (3)
	≥ 6 months, <1 year	11 (19)
	≥ 1, < 5 years	21 (36)
	≥ 5 years	24 (41)
Discussed in multidisciplinary team meeting	Yes	58 (100)
Treatment intention	Curative	21 (36)
	Local control	30 (51)
	Palliative	7 (12)
Radiotherapy planning technique	IMRT	56 (97)
	3D conformal	2 (3)
Treatment regimens applied	Surgery + 1.5 Gy × 30–40	21 (36)
	1.5 Gy × 40*	29 (50)
	1.5 Gy × 30	7 (12)
	1.5 Gy × 9**	1 (2)
Radiotherapy localization	Unilateral	43 (74)
	Bilateral	15 (26)
Concomitant systemic therapy	No	48 (83)
	Nivolumab	6 (10)
	Cisplatin	3 (5)
	Cetuximab	1 (2)
Radiotherapy intermission,	Yes	1 (2)
	No	57 (98)
Time as inpatient in days		12 [7] (0–62)

IMRT intensity modulated radiotherapy.

* Patients with dose painting (higher dose to part of the Gross Tumor Volume) (n = 8), and one patient had 44 fractions (66 Gy).

** Treatment prematurely stopped due to deterioration of disease.

Thirteen of 21 patients treated with curative intent had surgery followed by HFRT with ≥60 Gy to GTV (n = 1) and/ or CTV (n = 20). Eight of 30 patients with local control intent had postoperative HFRT with 45–60 Gy to the CTV, 20 had HFRT alone of ≥60 Gy and two stopped treatment before completion, due to own choice (45 Gy) and rapid progression of disease (13.5 Gy). Eight patients (with curative or local control intent) participated in the dose painting study and received 70–75 Gy to parts of the GTV [15]. Ten patients received concomitant systemic therapy (Table 2), six of these participated in a phase I study investigating HFRT in combination with immunotherapy (NCT03317327). Six of the seven patients who received palliative HFRT received 45 Gy to the GTV and/ or CTV and the last patient 60 Gy. He had salivary gland adenocarcinoma with inoperable perineural growth along the facial nerve. Only one patient had intermission of treatment, this was due to a need for re-planning.

### 3.2 HRQoL

Patients’ compliance was 100% from start, high throughout the follow-up period, but decreased from 94% at two years to 62% at three years post-treatment (Appendix A).

**Fig. 2:**
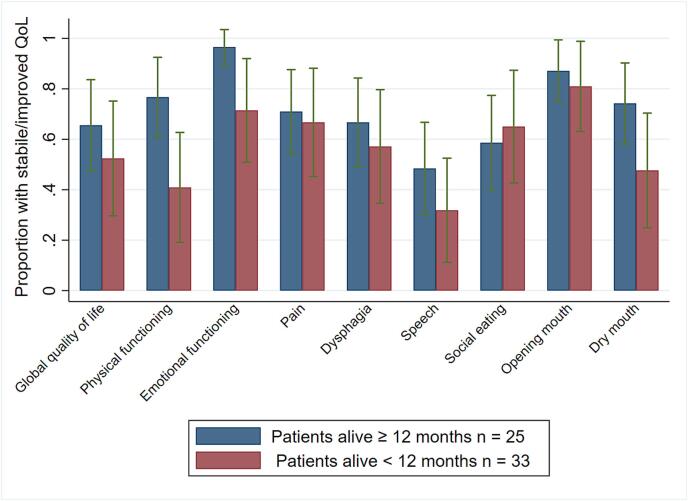
Proportion of patients with stable or improved HRQoL three months after start of treatment in patients who were alive 12 months or more vs patients who died within 12 months.

**Fig. 3:**
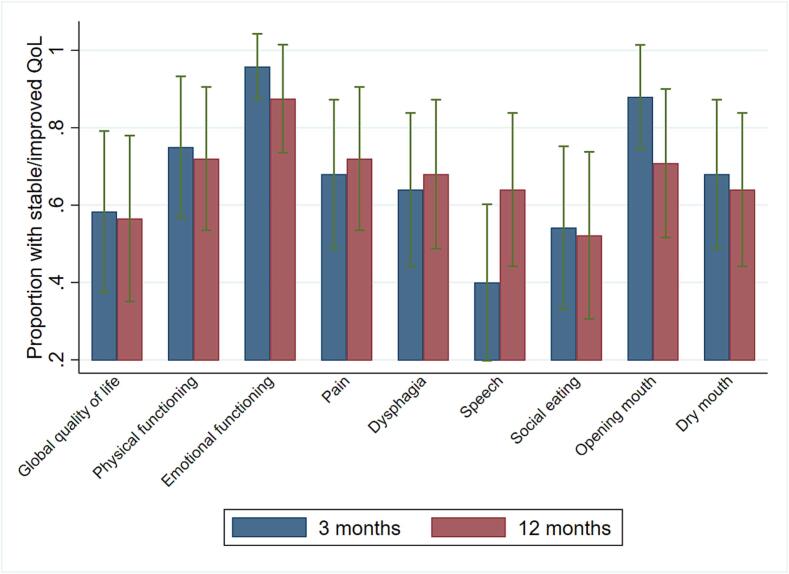
Proportion of patients with stable or improved HRQoL three and 12 months after treatment in patients alive at least 12 months.

There were no clinically significant change in the mean (95% CI) Global QoL −6.2 (-12.3, −0.04), p = 0.048 and H&N Pain 1.1 (-5.0, 7.3), p = 0.72 from pre-treatment to three-month follow-up (Table 3A). More than half of the patients reported stable or improved HRQoL at three months. They felt more ill and reported more speech problems than before treatment. Their social, emotional and cognitive functioning and other symptoms were stable. A higher proportion of patients that lived ≥ 12 months compared to those who lived < 12 months, had stable or improved physical and emotional functioning, and level of dry mouth at three months compared to pre-treatment (Fig. 2). At 12 months, there was a trend towards deteriorated mean (95% CI) Global QoL compared to pre-treatment −9.4 (-18.9, 0.1), p = 0.05, while the mean H&N Pain was unchanged 1.0 (−7.6, 9.6), p = 0.81 (Table 3B). Most patients who had stable/ improved HRQoL at three months who lived ≥12 months, also had stable/ improved HRQoL at 12 months (Fig. 3), except that fewer had speech problems and more patients had problems with opening the mouth. At three years, eight of 13 patients alive completed the questionnaires. They reported reduced functioning and Global QoL, stable H&N Pain, more dysphagia, problems with opening the mouth and problems with social eating, but less dry mouth and sticky saliva compared with pre-treatment (Appendix B).

**Table 3A:** Health-related quality of life in patients three months after treatment for relapse or second primary head and neck cancer and change from pre-treatment.

**Scales, items**	**Pre-treatment**^1^ n = 52	**3 months**^1^ n = 52	**Change from pre-treatment to 3 months**^2^ n = 52	**Stable or improved**^3^**HRQoL** n = 52
**EORTC QLQ-C30**	**Mean (SD)**	**Mean (SD)**	**Mean (95% CI)**	**n (%)**
Global quality of life	60 (21)	53 (21)**	−6.2 (-12.3 to −0.04)	30 (60)
Physical function	73 (21)	63 (26)	−9.6 (-14.5 to −4.6)	32 (62)
Role function	55 (33)	49 (36)	−6.7 (-16.5 to 3.0)	31 (60)
Emotional function	74 (19)	78 (19)**	3.9 (-2.5 to 10.2)	43 (86)
Cognitive function	81 (16)	75 (23)**	−6.3 (-11.5 to −1.2)	32 (64)
Social function	64 (25)	63 (29)**	−1.3 (-9.3 to 6.7)	33 (66)
Fatigue	48 (25)	51 (26)	2.7 (-4.8 to 10.1)	32 (62)
Nausea/vomiting	8 (16)	10 (14)	2.6 (-1.7 to 6.8)	42 (81)
Pain	37 (28)	36 (27)	−0.6 (-9.7 to 8.4)	36 (69)
Dyspnea	26 (31)	31 (32)**	4.7 (-3.5 to 12.8)	36 (70)
Insomnia	31 (32)	35 (34)	3.8 (-4.5 to 12.2)	36 (69)
Appetite loss	35 (35)	38 (39)	3.2 (-6.2 to 12.6)	39 (75)
Constipation	34 (32)	37 (36)**	2.7 (-8.5 to 13.8)	35 (70)
Diarrhea	12 (20)	17 (25)***	4.1 (-3.9 to 12.1)	39 (80)
Financial problems	13 (22)	23 (32)**	9.3 (1.7 to 17.0)	37 (74)
**EORTC QLQ-H&N35**	n = 53	n = 53	n = 53	n = 53
Pain	34 (22)	35 (23)*	1.1 (-5.0 to 7.3)	36 (69)
Swallowing	38 (29)	45 (30)**	7.1 (0.6 to 13.6)	32 (63)
Senses problems	34 (31)	39 (31)*	5.1 (-1.1 to 11.4)	32 (63)
Speech problems	**28 (25)**	**39 (31)**	**10.8 (3.9 to 17.7)**	22 (42)
Social eating	40 (29)*	46 (30)****	5.6 (-2.3 to 13.7)	30 (61)
Social contact	17 (20)	21 (24)	3.6 (-2.4 to 9.6)	39 (74)
Sexuality	47 (36)**	52 (39)***	4.7 (-5.4 to 14.7)	28 (56)
Teeth	27 (36)	22 (32)**	−5.2 (-15.0 to 4.6)	39 (76)
Opening mouth	51 (38)	44 (33)*	−7.1 (-15.9 to 1.8)	44 (85)
Dry mouth	56 (35)	62 (32)*	6.4 (-2.6 to 15.4)	33 (64)
Sticky saliva	56 (36)	63 (33)**	7.7 (-0.7 to 16.0)	36 (71)
Coughed	**27 (29)**	38 (27)**	**10.5 (4.1 to 16.8)**	33 (65)
Felt ill	**26 (27)**	38 (34)*	**11.5 (1.5 to 21.6)**	32 (63)
Pain killers	75 (43)	72 (45)	−3.8 (-17.0 to 9.5)	48 (91)
Nutritional supplements	65 (48)	75 (44)*	9.6 (-3.0 to 22.2)	44 (86)
Feeding tube	31 (47)	44 (50)*	13.5 (-1.2 to 28.1)	41 (80)
Weight loss	35 (48)	27 (45)**	−7.8 (-24.5 to 8.9)	44 (88)

CI = confidence interval, SD = standard deviation.

Missing values: *one missing, **two missing, ***three missing, ****four missing.

Bold: Clinically and statistically significant changes.

1 High score imply high level of functioning and high level of symptoms.

2 Negative change values (calculated as scores at three months – pretreatment) are decrease in symptom and reduced functioning score.

3 Stable (change within ± 10), improvement of functioning scores change > 10 and improvement in symptoms change < -10.

**Table 3B:** Health-related quality of life in patients 12 months after treatment for relapse or second primary head and neck cancer and change from pre-treatment.

**Scales, items**	**Pre-treatment**^1^ n = 25	**12 months**^1^ n = 25	**Change from pre-treatment to 12 months**^2^ n = 25	**Stable or improved**^3^ **HRQoL** n = 25
**EORTC QLQ-C30**	**Mean (SD)**	**Mean (SD)**	**Mean (95% CI)**	**n (%)**
Global quality of life	61 (20)**	51 (22)**	−9.4 (-18.9 to 0.1)	13 (56)
Physical function	73 (20)	70 (22)	−2.6 (-8.8 to 3.6)	18 (72)
Role function	56 (32)	53 (28)	−3.3 (-14.3 to 7.7)	17 (68)
Emotional function	72 (20)*	77 (22)*	4.6 (-1.8 to 11.0)	21 (88)
Cognitive function	78 (17)	74 (23)*	−4.2 (-11.7 to 3.4)	16 (67)
Social function	64 (24)	56 (30)*	−7.6 (-18.6 to 3.3)	12 (50)
Fatigue	49 (27)	46 (26)	−2.7 (-11.7 to 6.3)	16 (64)
Nausea/vomiting	7 (14)	10 (17)	3.3 (-6.2 to 12.9)	18 (72)
Pain	41 (30)	40 (30)	−0.7 (-12.5 to 11.2)	14 (56)
Dyspnea	27 (32)	33 (33)	6.7 (-3.1 to 16.4)	17 (68)
Insomnia	33 (32)	32 (26)	−1.3 (-13.6 to 10.9)	17 (68)
Appetite loss	31 (32)	31 (38)	0.0 (-15.9 to 15.9)	20 (80)
Constipation	26 (26)*	36 (28)*	9.7 (-3.7 to 23.2)	14 (58)
Diarrhea	13 (22)*	18 (28)*	5.5 (-2.4 to 13.5)	20 (83)
Financial problems	20 (26)**	20 (33)**	0.0 (-12.3 to 12.3)	19 (82)
**EORTC QLQ-H&N35**	n = 25	n = 25	n = 25	n = 25
Pain	32 (20)	33 (24)	1.0 (-7.6 to 9.6)	18 (72)
Swallowing	35 (34)	43 (31)	8.0 (-2.2 to 18.2)	17 (68)
Senses problems	35 (30)	35 (28)	−0.7 (-9.2 to 7.9)	19 (76)
Speech problems	34 (29)	36 (26)	2.0 (-9.4 to 13.4)	16 (64)
Social eating	32 (27)**	39 (20)**	8.0 (-3.3 to 19.2)	12 (52)
Social contact	15 (16)*	21 (22)*	5.1 (-3.5 to 13.8)	17 (68)
Sexuality	49 (35)*	51 (46)*	1.4 (-14.8 to 17.6)	14 (58)
Teeth	25 (32)	35 (35)	9.3 (-3.5 to 22.2)	16 (64)
Opening mouth	50 (35)*	53 (37)*	2.8 (-13.3 to 18.8)	17 (68)
Dry mouth	53 (37)	61 (34)	8.0 (-5.9 to 21.9)	16 (64)
Sticky saliva	52 (37)	43 (33)	−9.3 (-27.3 to 8.7)	17 (68)
Coughed	24 (30)	29 (29)	5.3 (-4.9 to 15.6)	19 (76)
Felt ill	25 (26)	25 (34)	0.0 (-14.9 to 14.9)	18 (72)
Pain killers	71 (46)*	63 (49)*	−8.3 (-29.6 to 12.9)	22 (92)
Nutritional supplements	63 (49)*	79 (41)*	16.7 (-43.6 to 10.2)	18 (75)
Feeding tube	38 (49)*	38 (49)*	0.0 (-17.6 to 17.6)	22 (92)
Weight loss	29 (46)*	17 (38)*	−12.5 (-41.2 to 16.2)	20 (83)

CI = confidence interval, SD = standard deviation.

Missing values: *one missing, **two missing.

Bold: Clinically and statistically significant changes.

1 High score imply high level of functioning and high level of symptoms.

2 Negative change values (calculated as scores at 12 months – pretreatment) are decrease in symptom and reduced functioning score.

3 Stable (change within ± 10), improvement of functioning scores change > 10 and improvement in symptoms change < -10.

### 3.3 Toxicity and time in hospital

At EOT, 41 (71%) patients experienced serious toxicity (≥grade 3 CTCAE score) (Table 4), but 25 of them suffered from serious toxicity before the current treatment as well. The most common were severe mucositis, dysphagia, anorexia and fatigue. Nine of the 25 patients with serious dysphagia at EOT, and eight of the 18 patients with serious dysphagia at three months, had unchanged level from pre-treatment. At three months, there were fewer patients with severe mucositis, dysphagia, anorexia and fatigue (Table 4) than at EOT when corrected for dropouts (data not shown). At six months, three patients had osteoradionecrosis that needed surgery (Appendix C). Three patients died of carotid blowout; one, three and six months after HFRT of 60 Gy. One had tumour invasion of the artery due to local relapse in the oropharynx more than four years after primary treatment. The other two had complications of re-irradiation for SP (T2 oropharyngeal and T4b laryngeal cancer more than five years after primary treatment). The cumulative near maximum dose (D1cc) to the carotid artery in these three cases were 110.7 Gy, 127.6 Gy and 120.4 Gy. Seventeen patients completed treatment as outpatients while 41 inpatients had a median stay of seven days. At EOT, 11 patients were discharged to the local hospital or nursing home while 47 went home.

**Table 4:** Acute and late toxicity; number of patients (%) with ≥ grade 3 CTCAE score.

	**Pre-treatment** n = 58	**End of treatment** n = 58	**3 months** n = 54	**12 months** n = 27	**36 months** n = 10
	n (%)	n (%)	n (%)	n (%)	n (%)
Mucositis	0 (0)	9 (15)	2 (4)	0 (0)	1 (10)
Dysphagia	12 (21)	25 (43)	18 (33)	9 (30)	4 (40)
Taste alteration	0 (0)	0 (0)	0 (0)	0 (0)	0 (0)
Edema	1 (2)	2 (3)	0 (0)	0 (0)	0 (0)
Hoarseness	5 (9)	6 (10)	4 (7)	5 (19)	3 (30)
Dermatitis	0 (0)	2 (3)	1 (2)	0 (0)	0 (0)
Xerostomia	5 (9)	9 (15)	6 (11)	1 (4)	0 (0)
Fistula	0 (0)	0 (0)	0 (0)	0 (0)	0 (0)
Trismus	3 (5)	2 (3)	2 (4)	4 (15)	2 (20)
Osteonecrosis	0 (0)	0 (0)	0 (0)	1 (4)	0 (0)
Indurations/Fibrosis	2 (4)	8 (14)	6 (11)	6 (22)	5 (50)
Fatigue	4 (7)	11 (19)	6 (11)	5 (19)	2 (20)
Anorexia	3 (5)	11 (19)	6 (11)	5 (19)	1 (10)
Nausea	0 (0)	2 (3)	0 (0)	1 (4)	0 (0)
Constipation	0 (0)	0 (0)	1 (2)	0 (0)	0 (0)
Pain (local)	3 (5)	7 (12)	4 (7)	6 (22)	0 (0)
Pain (other)	1 (2)	2 (3)	0 (0)	0 (0)	2 (20)
Infection	1 (2)	3 (5)	4 (7)	2 (7)	1 (10)
Cardiac	0 (0)	1 (1)	0 (0)	0 (0)	0 (0)
Pulmonary	2 (4)	1 (1)	3 (6)	2 (7)	2 (20)
Any toxicity	28 (48)	41 (71)	32 (58)	15 (56)	7 (70)

### 3.4 Survival and disease status

**Fig. 4:**
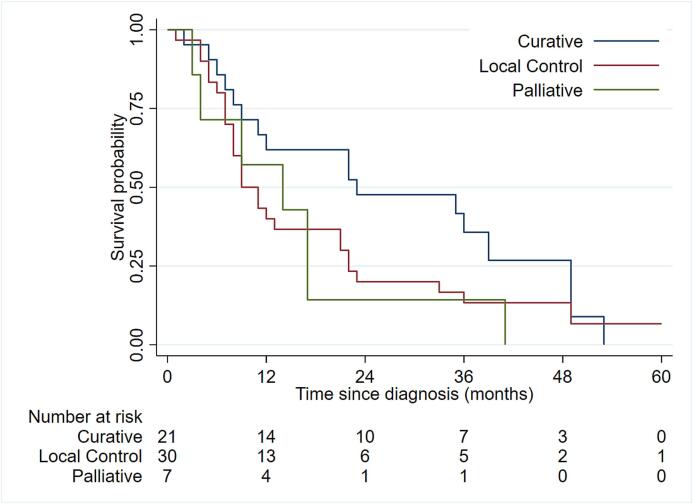
Overall survival for patients treated with curative, local control or palliative intent during five years of follow-up.

Median survival (range) for the total group was 12 months (1–66). For patients with curative, local control and palliative intent, median survival (range) was 23 (2–53), 10 (1–66) and 14 (3–41) months, respectively (Fig. 4). Thirty-one patients (52%) were alive at 12 months. At three years, 13 (25%) of the 51 patients treated with curative or local control intent were alive. For the 21 patients treated with curative intent, there were no observed difference in median survival between those who had surgery and HFRT (22 months) and those treated with HFRT alone (23 months). Thirty-two patients died of the index cancer, eight of other disease or other causes, three with treatment complications and six of unknown reasons.

**Fig. 5:**
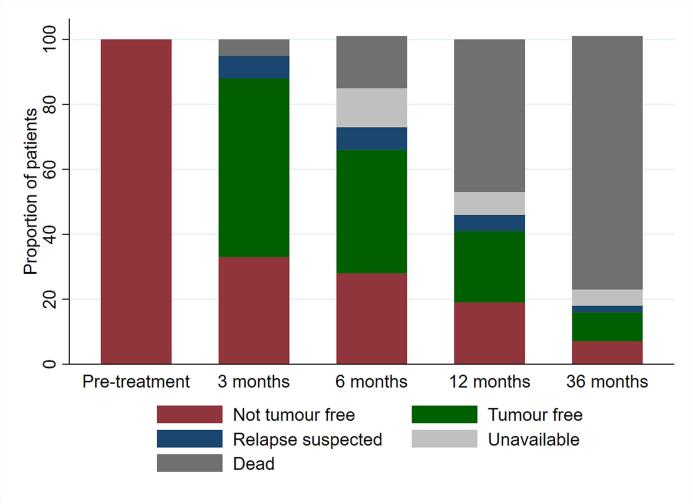
Disease status at pre-treatment and during follow-up for patients eligible for hyperfractionated re-irradiation n = 58.

Disease status recorded at three, six, 12 and 36 months are presented in Fig. 5. The proportion of disease-free patients of those alive and available for assessment at three and 12 months follow-up were 58% (32/55) and 48% (13/27), respectively. Within the subgroup of patients who were not disease free at three months (n = 19), 11 patients reported stable or improved HRQoL compared to pre-treatment, and the mean change in Global QoL −4.9 (−16. 9, 7.1) was not significantly reduced. Disease and clinical status of patients alive at 12 and 36 months are displayed in Table 5, and their pre-treatment characteristics in Appendix D. Patients alive at three years were somewhat younger, more often females without comorbidity than the total patient sample, but had similar disease status, tumour location, histology and WHO performance status. The majority of patients alive at one and three years had received ≥60 Gy.

**Table 5:** Patients tumour and clinical status at 12 and 36 months after start of treatment for relapse of head and neck cancer or second primary in the head and neck area.

**Characteristics**	**12 months**	**36 months**
	n = 27*	n = 10**
	n (%)	n (%)
**Tumour status**		
Tumour free	13 (48)	5 (50)
Relapse suspected	3 (11)	1 (10)
Not tumour free	11 (41)	4 (40)
**WHO performance status**		
0–1	17 (63)	5 (50)
2	8 (30)	4 (40)
3	0 (0)	1 (10)
Missing	2 (7)	0 (0)
**Pain medication**	18 (67)	6 (60)
**Nasogastric tube**	1 (4)	0 (0)
**Percutaneous endoscopic gastrostomy**	8 (30)	6 (60)
**Tracheostomy**	5 (19)	3 (30)

*4 patients were not available for clinical evaluation at 12 months follow-up.

**3 patients were not available for clinical evaluation 36 months after start of treatment.

## 4 Discussion

This first prospective longitudinal study of potential HRQoL benefit of HFRT in patients with recurrent or SP HNC demonstrate that most patients had maintained or improved HRQoL at three and 12 months after treatment. This supports that HFRT can be worthwhile despite the poor prognosis and high prevalence of toxicity at EOT. HFRT with three to four weeks of treatment without intermission was feasible as all but two patients completed treatment as planned; many as outpatients or with a short stay in hospital.

Our results are in line with two studies of 15 and 17 patients reporting maintained HRQoL one year after treatment [6], [21]. Although there was a trend towards deteriorated mean Global QoL at 12 months in our study, most patients reported stable or improved level of HRQoL in most of the domains. In a study of adjuvant stereotactic re-irradiation (SBRT) following salvage surgery, 56% of the patients reported improved/stable HRQoL at median six months follow-up [22]. Another study reported improved/stable HRQoL in patients receiving SBRT to the skull base [23], while after SBRT to the neck, patients had increased dysphagia [23]. Due to the different treatment modalities applied, the results are not directly comparable with ours.

As expected, the proportion of patients with acute grade ≥3 toxicity at EOT was high. The prevalence of grade 3 mucositis was 15%, which is in accordance with others [9]. Transient worsening of symptoms at EOT is often accepted while maintained or increased level of toxicity during follow-up may question the benefit of treatment. One third of our patients experienced late dysphagia grade ≥3, which is higher compared to others [24]. However, nearly half of these patients had grade ≥3 dysphagia at pre-treatment which might be part of the explanation. Carotid blow-out can occur with tumour invasion of the vessel as seen in one of our patients, but is also a feared complication of treatment as observed in two other patients. Whether the risk might be increased with HFRT compared to conventional fractionation has been questioned [25]. Heterogeneity in the studies reviewed in literature and the role of concomitant treatment applied makes it difficult to draw conclusions. The reported rates of carotid blow-out are 0–17% with the highest risk reported in patients treated with conventional fractionation for nasopharyngeal cancer [26].

In patients with palliative treatment intent, the 14 months median survival is comparable with HNC patients treated with palliative immunotherapy [27]. Immunotherapy imply fewer hospital attendances and lower toxicity rates and may be a good alternative treatment for future palliative HNC patients. On the other hand, the median survival of 23 months in patients with curative treatment intent, is a confirmation of the poor prognosis of these patients described in the literature [3]. The large differences reported in median survival of re-irradiated patients, from six to 28 months [28], [29], [30], [31], may be explained by the patient heterogeneity. Postoperative re-irradiation has been associated with improved survival versus primary re-irradiation [3], [8]. We were therefore surprised that there was no difference in median survival between patients with curative intent who had surgery followed by HFRT vs HFRT alone. However, this might be explained by the patient heterogeneity and the small sample size of this study.

This study supports that for selected patients where the treatment is given with curative or life prolonging intent, HFRT of 1.5 Gy × 2 × 20 is feasible and offer maintained HRQoL for most patients despite the high toxicity rate. Our finding that all but one patient who were alive three years after start of treatment received at least 60 Gy, supports this being the recommended dose level to achieve local control. The dose response in terms of loco-regional control and survival has also been supported by others [32], [33]. Tumour control is necessary to achieve long term survival, and is essential for maintaining HRQoL as the tumour may affect basic functions such as opening of the mouth, swallowing and speech. Although the proportion of patients alive decreased during follow-up, the high compliance in the PROs among those alive help to picture the clinical reality for these patients. However, the few patients who were not available for assessments may have been seriously ill and their drop-outs could potentially bias the HRQoL results, particularly at three years. To further improve the survival and reduce the toxicity and thereby improve patients’ HRQoL, dose painting with increased dose to the areas of high tumour activity that may improve tumour control or concomitant immunotherapy could be considered as potential options [15], [34]. The possible benefit of proton re-irradiation is currently being studied [35] and this study will serve as historical control to a future proton re-irradiation study planned in Norway.

Due to low recruitment rate, we were unable to reach the target patient number within the predefined recruitment period of four years. The small sample and patient population heterogeneity limit our ability to test prognostic factors to improve patient selection. Also, the variety of treatment combinations applied limits our ability to evaluate the outcomes. The study design does not allow for comparison of outcome with other treatments. Even though randomised studies are warranted, they are difficult to perform due to few eligible patients and lack of consensus on the preferred treatment between centres. The study is strengthened by the prospective design and research questions decided upfront in collaboration with patient representatives. The high compliance, use of validated questionnaires and low frequency of missing data give a reliable view of patients’ HRQoL. Hopefully, the results will inform clinicians and patients about the expected outcome of treatment, improve shared decision-making and improve the clinical follow-up and palliative care. In Norway, 60% of HNC patients are treated at our institution. As we used consecutive enrolment of patients and wide inclusion criteria, we believe our results are representative for HNC patients with relapse and SP in Norway and probably also in other countries.

## 5 Conclusion

This study demonstrate that despite limited overall survival and serious toxicity in various patients after HFRT without intermission for recurrent or SP HNC, most patients reported maintained HRQoL at three and 12 months after treatment. Long-term survival can be achieved in a limited proportion of the patients.

## Funding

Department of Oncology, Oslo University Hospital, provided internal research-funding for JM Moan, PhD 50% position over 4 years.

## Ethics approval

Approved by The Regional Committees for Medical and Health Research Ethics (reference number **2015/118**), the local protocol committee (date 23/04/2015/reference 2015-23) and the Oslo University Hospital privacy office (date 30/03/2015).

## Consent to participate

All participants provided informed consent before inclusion in the study.

## Declaration of Competing Interest

The authors declare that they have no known competing financial interests or personal relationships that could have appeared to influence the work reported in this paper.
