# Supplementary material for: Impact of hyperfractionated re-irradiation on quality of life in patients with recurrent or second primary head and neck cancer, a prospective single institutional study
Source: Clin Transl Radiat Oncol. 2023 Jun 22;42:100654. doi: 10.1016/j.ctro.2023.100654 (PMC10319815; doi:10.1016/j.ctro.2023.100654)
Supplement: Supplementary data 1 [file mmc1.docx]

**Appendix A. Health-related quality of life for patients alive at each time point**

|  | **Mean score (SD)** | | | | | | | | |
| --- | --- | --- | --- | --- | --- | --- | --- | --- | --- |
| EORTC QLQ-C30  QoL, Scales, items  Compliance (% of patients alive) | **Baseline**  n = 58  (100%) | **End of treatment**  n = 55  (95%) | 3 months  n = 52  (95%) | 6 months  n = 40  (82%) | 12 months  n = 25  (81%) | 18 months  n = 19  (76%) | 24 months  n = 16  (94%) | 30 months  n = 14  (82%) | 36 months  n =8  (62%) |
| Global quality of life | 59 (22) | 54 (19) | 53 (21)** | 55 (24) | 51 (22)* | 48 (21)* | 53 (17)* | 51 (21) | 50 (29) |
| Physical function | 72 (23) | 65(22) | 63 (26) | 71 (22) | 70 (22) | 66 (20) | 71 (22) | 72 (26) | 65 (32) |
| Role function | 55 (33) | 47 (32) | 49 (36) | 61 (33) | 53 (28) | 50 (34) | 63 (26) | 57 (34) | 46 (35) |
| Emotional function | 74 (21) | 80 (17) | 78 (19)** | 81 (19) | 77 (22)* | 75 (18)* | 80 (23)* | 79 (24) | 66 (29) |
| Cognitive function | 82 (16) | 74 (22) | 75 (23)** | 80 (20) | 74 (23)* | 71 (25)* | 71 (26)* | 79 (24) | 73 (28) |
| Social function | 65 (25) | 63 (29) | 63 (29)** | 60 (32) | 56 (30) * | 56 (33)* | 54 (30)* | 51 (27) | 50 (29) |
| Fatigue | 47 (25) | 57 (24) | 51 (26) | 47 (26) | 46 (26) | 53 (22) | 46 (25) | 44 (26) | 47 (29) |
| Nausea/vomiting | 9 (16) | 15 (21) | 10 (14) | 5 (11) | 10 (17) | 12 (19) | 7 (15) | 10 (14) | 4 (12) |
| Pain | 39 (29) | 46 (27) | 36 (27) | 31 (26) | 40 (30) | 46 (34) | 33 (29) | 36 (35) | 38(419 |
| Dyspnea | 28 (31) | 27 (29) | 31 (32)** | 27 (31) | 33 (33) | 46 (37) | 38 (38) | 24 (36) | 33 (47) |
| Insomnia | 32 (31) | 30 (29) | 35 (34) | 23 (27) | 32 (26) | 33 (27) | 29 (27) | 36 (27) | 42 (30) |
| Appetite loss | 36 (36) | 52 (37) | 38 (39) | 31 (37) | 31 (38) | 37 (36)* | 33 (37) | 38 (39) | 25 (39) |
| Constipation | 32 (31) | 46 (33) | 37 (36)** | 30 (35) | 36 (28)* | 37 (30)* | 33 (33)* | 33 (35) | 46 (35) |
| Diarrhea | 11 (19) | 19 (31)* | 17 (25)*** | 14 (23) | 18 (28)* | 24 (30)* | 11 (21)* | 12 (21) | 13 (17) |
| Financial problems | 11 (21) | 13 (25)* | 23 (32)** | 21 (31) | 20 (33)** | 9 (19)* | 18 (33)* | 21 (34) | 21 (25) |
| EOTC QLQ-H&N35 |  |  |  |  |  |  |  |  |  |
| Pain | 34 (23) | 47 (26) | 35 (23) | 36 (20) | 33 (24) | 32 (27) | 39 (31) | 33 (32) | 25 (25) |
| Swallowing | 40 (29) | 52 (29) | 45 (30)* | 47 (31) | 43 (31) | 48 (32)* | 51 (29) | 58 (34) | 67 (32) |
| Senses problems | 32 (30) | 43 (31) | 39 (31) | 36 (32) | 35 (28) | 39 (26) | 45 (32) | 43 (29) | 58 (30) |
| Speech problems | 30 (26) | 40 (30) | 39 (31) | 35 (30) | 36 (26) | 40 (27) | 35 (28) | 37 (27) | 49 (30) |
| Social eating | 43 (29)* | 52 (31) | 47 (30)** | 41 (31) | 42 (30)* | 46 (31)** | 52 (29)* | 60 (37) | 56 (35) |
| Social contact | 17 (20) | 22 (25) | 21 (24) | 17 (19) | 21 (22)* | 23 (20)* | 23 (24) | 26 (22) | 29 (27) |
| Sexuality | 49 (36)** | 57 (41)* | 52 (39)** | 48 (41)** | 50 (46)* | 56 (45)* | 58 (41) | 55 (44) | 74 (38) |
| Teeth | 27 (36)* | 29 (38) | 22 (32)** | 28 (32)* | 35 (35) | 21 (25) | 29 (30) | 43 (40) | 42 (39) |
| Opening mouth | 50 (38) | 53 (37) | 44 (33) | 54 (35) | 53 (37)* | 51 (39) | 51 (30)* | 55 (38) | 62 (38) |
| Dry mouth | 57 (36) | 67 (35)* | 62 (32) | 56 (36) | 61 (34) | 53 (37) | 54 (29) | 60 (42) | 29 (42) |
| Sticky saliva | 57 (37) | 74 (29) | 63 (33) | 63 (33)* | 43 (33) | 58 (31) | 42 (29) | 38 (29) | 42 (35) |
| Coughed | 30 (31) | 39 (31) | 38 (27)* | 34 (30) | 29 (29) | 35 (34) | 33 (38) | 41 (41) | 38 (42) |
| Felt ill | 26 (27) | 36 (32) | 38 (34)* | 29 (32) | 25 (34)* | 30 (31) | 29 (24) | 33 (35) | 29 (28) |
| Pain killers | 74 (44) | 80 (40) | 72 (45) | 58 (50) | 63 (46) | 67 (49)* | 47 (52)* | 57 (51) | 50 (53) |
| Nutritional supplements | 69 (47) | 85 (36) | 72 (45)* | 75 (44) | 79 (41) | 78 (43)* | 67 (49)* | 71 (47) | 75 (46) |
| Feeding tube | 31 (47) | 40 (49) | 44 (50) | 43 (50)* | 38 (49) | 44 (51)* | 33 (49)* | 43 (51) | 75 (46) |
| Weight loss | 36 (48) | 61 (49)* | 27 (45)* | 28 (46)* | 17 (38) | 17 (38)* | 27 (46)* | 57 (51) | 25 (46) |

High score imply high level of functioning and high level of symptoms.

Missing values: *one missing, **two missing , ***three missing

Bold: Clinical significant change
