# Supplementary material for: Impact of hyperfractionated re-irradiation on quality of life in patients with recurrent or second primary head and neck cancer, a prospective single institutional study
Source: Clin Transl Radiat Oncol. 2023 Jun 22;42:100654. doi: 10.1016/j.ctro.2023.100654 (PMC10319815; doi:10.1016/j.ctro.2023.100654)
Supplement: Supplementary data 2 [file mmc2.docx]

|  | **Baseline** | | | |  | | **End of Treatment** | | | | |  | **3 months** | | | | |  | **6 months** | | | | |  | **12 months** | | | | |  | **36 months** | | | | |
| --- | --- | --- | --- | --- | --- | --- | --- | --- | --- | --- | --- | --- | --- | --- | --- | --- | --- | --- | --- | --- | --- | --- | --- | --- | --- | --- | --- | --- | --- | --- | --- | --- | --- | --- | --- |
|  | n = 58 | | | |  | | n = 58 | | | | |  | n= 54 | | | | |  | n = 41 | | | | |  | n = 27 | | | | |  | n- = 10 | | | | |
| CTCAE grade | **0** | **1** | **2** | **3** | **4** |  | **0** | **1** | **2** | **3** | **4** |  | **0** | **1** | **2** | **3** | **4** |  | **0** | **1** | **2** | **3** | **4** |  | **0** | **1** | **2** | **3** | **4** |  | **0** | **1** | **2** | **3** | **4** |
| Mucositis | 54 | 4 |  |  |  |  | 14 | 14 | 20 | 9 |  |  | 30 | 12 | 9 | 2 |  |  | 33 | 5 |  |  |  |  | 22 | 5 |  |  |  |  | 8 | 1 | 1 |  |  |
| Dysphagia | 21 | 12 | 13 | 5 | 7 |  | 5 | 7 | 21 | 17 | 8 |  | 6 | 8 | 22 | 14 | 4 |  | 3 | 7 | 17 | 10 | 3 |  | 2 | 6 | 10 | 5 | 4 |  | 2 | 1 | 3 | 3 | 1 |
| Taste alteration | 25 | 21 | 12 |  |  |  | 9 | 24 | 22 |  |  |  | 10 | 28 | 14 |  |  |  | 11 | 19 | 9 |  |  |  | 7 | 13 | 7 |  |  |  | 4 | 3 | 2 |  |  |
| Edema | 36 | 19 | 2 | 1 |  |  | 29 | 20 | 6 | 1 | 1 |  | 25 | 20 | 6 |  |  |  | 17 | 14 | 5 | 1 |  |  | 21 | 4 | 1 |  |  |  | 7 | 1 | 2 |  |  |
| Hoarseness | 33 | 16 | 4 | 2 | 3 |  | 22 | 19 | 9 | 1 | 5 |  | 21 | 20 | 9 | 1 | 3 |  | 17 | 10 | 10 | 1 | 3 |  | 11 | 6 | 5 | 2 | 3 |  | 5 | 1 | 1 | 2 | 1 |
| Dermatitis | 53 | 4 | 1 |  |  |  | 6 | 43 | 6 | 2 |  |  | 37 | 13 | 1 | 1 |  |  | 34 | 5 |  |  |  |  | 23 | 3 |  |  |  |  | 10 |  |  |  |  |
| Xerostomia | 14 | 29 | 10 | 5 |  |  | 9 | 25 | 14 | 9 |  |  | 3 | 25 | 19 | 6 |  |  | 5 | 20 | 12 | 2 |  |  | 3 | 14 | 9 | 1 |  |  |  | 4 | 6 |  |  |
| Fistula | 56 |  | 2 |  |  |  | 58 |  |  |  |  |  | 53 | 1 |  |  |  |  | 39 | 1 | 1 |  |  |  | 26 | 1 |  |  |  |  | 9 | 1 |  |  |  |
| Trismus | 23 | 24 | 8 | 3 |  |  | 16 | 27 | 12 | 2 |  |  | 17 | 18 | 16 | 2 |  |  | 12 | 15 | 10 | 4 |  |  | 8 | 7 | 8 | 4 |  |  | 2 | 4 | 1 | 2 |  |
| Osteonecrosis | 58 |  |  |  |  |  | 58 |  |  |  |  |  | 53 | 1 |  |  |  |  | 37 | 1 |  | 3 |  |  | 25 | 1 | 1 |  |  |  | 9 | 1 |  |  |  |
| Indurations/Fibrosis | 23 | 13 | 20 | 2 |  |  | 13 | 14 | 22 | 8 |  |  | 10 | 11 | 24 | 6 |  |  | 8 | 7 | 15 | 5 |  |  | 4 | 7 | 9 | 6 |  |  |  | 5 | 5 |  |  |
| Bleeding | 54 | 3 | 1 |  |  |  | 38 | 18 | 1 |  |  |  | 47 | 6 |  |  |  |  | 31 | 8 |  |  |  |  | 20 | 7 |  |  |  |  | 8 | 1 | 1 |  |  |
| Fatigue | 21 | 28 | 5 | 4 |  |  | 14 | 23 | 10 | 10 | 1 |  | 14 | 22 | 12 | 6 |  |  | 9 | 16 | 11 | 5 |  |  | 5 | 10 | 7 | 4 | 1 |  | 2 | 3 | 3 | 2 |  |
| Anorexia | 34 | 4 | 12 | 8 |  |  | 23 | 12 | 12 | 11 |  |  | 17 | 20 | 11 | 6 |  |  | 18 | 16 | 6 | 1 |  |  | 12 | 6 | 4 | 5 |  |  | 3 | 4 | 2 | 1 |  |
| Nausea | 53 | 3 | 2 |  |  |  | 38 | 12 | 6 | 2 |  |  | 43 | 9 | 2 |  |  |  | 38 | 2 |  | 1 |  |  | 22 | 4 | 1 |  |  |  | 10 |  |  |  |  |
| Constipation | 32 | 17 | 9 |  |  |  | 18 | 24 | 14 | 1 |  |  | 25 | 15 | 13 |  |  |  | 26 | 9 | 6 |  |  |  | 14 | 7 | 6 |  |  |  | 3 | 5 | 2 |  |  |
| Pain (local) | 17 | 17 | 21 | 3 |  |  | 8 | 18 | 24 | 7 |  |  | 13 | 24 | 13 | 4 |  |  | 13 | 20 | 6 | 2 |  |  | 6 | 11 | 4 | 6 |  |  | 3 | 5 | 2 |  |  |
| Pain (other) | 44 | 6 | 7 | 1 |  |  | 43 | 9 | 4 | 2 |  |  | 40 | 8 | 5 |  |  |  | 30 | 6 | 3 | 2 |  |  | 16 | 6 | 5 |  |  |  | 7 | 1 | 2 |  |  |
| Infection | 53 | 1 | 3 | 1 |  |  | 52 | 1 | 2 | 3 |  |  | 48 | 1 | 1 | 4 |  |  | 34 | 1 | 3 | 3 |  |  | 24 | 1 | 2 |  |  |  | 8 | 1 | 1 |  |  |
| Cardiac | 54 | 1 | 3 |  |  |  | 52 | 3 | 2 | 1 |  |  | 51 | 1 | 2 |  |  |  | 37 | 3 | 1 |  |  |  | 26 | 1 |  |  |  |  | 10 |  |  |  |  |
| Pulmonary | 52 | 2 | 2 | 2 |  |  | 55 | 2 |  | 1 |  |  | 46 | 2 | 3 | 3 |  |  | 37 | 3 | 1 |  |  |  | 23 | 1 | 1 | 2 |  |  | 7 | 1 | 2 |  |  |

**Appendix C.** **Acute and late toxicity; number of patients with grade 0 - 4 CTCAE score**
