# Supplementary material for: Impact of hyperfractionated re-irradiation on quality of life in patients with recurrent or second primary head and neck cancer, a prospective single institutional study
Source: Clin Transl Radiat Oncol. 2023 Jun 22;42:100654. doi: 10.1016/j.ctro.2023.100654 (PMC10319815; doi:10.1016/j.ctro.2023.100654)
Supplement: Supplementary data 3 [file mmc3.docx]

**Appendix B: Health-related quality of life at three years compared to baseline for patients who filled in both questionnaires (n = 8)**

| EORTC QLQ-C30 | Mean score (SD) | |
| --- | --- | --- |
| QoL, Scales, items | Baseline n = 8 | 36 months n =8 |
| Global quality of life | 65 (28) | **50 (29)** |
| Physical function | 78 (20) | **65 (32)** |
| Role function | 65 (29) | **46 (35)** |
| Emotional function | 81 (11) | **66 (29)** |
| Cognitive function | 77 (18) | 73 (28) |
| Social function | 71 (29) | **50 (29)** |
| Fatigue | 48 (25) | 47 (29) |
| Nausea/vomiting | 6 (18) | 4 (12) |
| Pain | 25 (25) | **38 (41)** |
| Dyspnea | 29 (37) | 33 (47) |
| Insomnia | 29 (33) | **42 (30)** |
| Appetite loss | 29 (28) | 25 (39) |
| Constipation | 29 (27) | **46 (35)** |
| Diarrhea | 8 (24) | 13 (17) |
| Financial problems | 13 (17) | 21 (25) |
| EOTC QLQ-H&N35 |  |  |
| Pain | 29 (22) | 25 (25) |
| Swallowing | 54 (36) | **67 (32)** |
| Senses problems | 33 (25) | **58 (30)** |
| Speech problems | 46 (29) | 49 (30) |
| Social eating | 45 (40) | **56 (35)** |
| Social contact | 14 (14) | **29 (27)** |
| Sexuality | 50 (29) | **74 (38)** |
| Teeth | 25 (39) | **42 (39)** |
| Opening mouth | 33 (31) | **62 (38)** |
| Dry mouth | 46 (35) | **29 (42)** |
| Sticky saliva | 58 (39) | **42 (35)** |
| Coughed | 38 (38) | 38 (42) |
| Felt ill | 17 (18) | **29 (28)** |
| Pain killers | 75 (46) | **50 (53)** |
| Nutritional supplements | 63 (52) | **75 (46)** |
| Feeding tube | 75 (46) | 75 (46) |
| Weight loss | 25 (46) | 25 (46) |

Bold: clinical significant change

High score imply high level of functioning and high level of symptoms.
