# Supplementary material for: Impact of hyperfractionated re-irradiation on quality of life in patients with recurrent or second primary head and neck cancer, a prospective single institutional study
Source: Clin Transl Radiat Oncol. 2023 Jun 22;42:100654. doi: 10.1016/j.ctro.2023.100654 (PMC10319815; doi:10.1016/j.ctro.2023.100654)
Supplement: Supplementary data 4 [file mmc4.docx]

**Appendix D. Baseline characteristics of patients alive at 1 and 3 years**

| **Characteristics at baseline** | Patients that survived at least 12 months  N (%) 31 (100 %) | Patients that survived at least 36 months  N = 13 |
| --- | --- | --- |
| **Age** Mean (range) | 62 (22-84) | 62 (38-84) |
| **Gender**  Male/female | 20 (65) / 11(36) | 8/5 |
| **Disease status**  Recurrent disease  Second primary | 21 (67)  10 (33) | 8 (62)  5 (38) |
| **Tumor location**  Oral cavity  Oropharynx  Hypopharynx  Nasopharynx  Larynx  Other | 11 (36)  8 (26)  4 (13)  1 (3)  4 (13)  3 (10) | 3 (23)  3 (23)  2 (15)  0 (0)  3 (23)  2 (15) |
| **Histology**  Squamous cell carcinoma  Adenocarcinoma  Adenoid cystic carcinoma  Other | 28 (90)  1 (3)  1(3)  1 (3) | 11 (85)  0 (0)  1 (8)  1 (8) |
| **WHO performance status**  0  1  2 | 11 (37)  15 (44)  5 (19) | 4 (31)  7 (54)  2 |
| Comorbidity  No  Yes | 17 (55)  14 (45) | 9 (69)  4 (31) |
| **Treatment**  Surgery + HFRT  HFRT | 13 (42)  18 (58) | 6 (46)  7 (54) |
| Total dose ≥ 60 Gy  Total dose 45 Gy | 27 (87)  4 (13) | 12 (92)  1 (8) |
